# Analysis of extrauterine growth retardation and related risk factors in 132 premature infants

**DOI:** 10.12669/pjms.38.6.5864

**Published:** 2022

**Authors:** Meng Sun, Jing Lu, Meng Sun, Yan Zheng, Qianqian Zhu, Caixia Liu

**Affiliations:** 1 Meng Sun, Department of Early Childhood Development Center, Zaozhuang Maternal and Child Health Hospital, Zaozhuang, Shandong Province 277100, P.R. China; 2 Jing Lu, Department of Children’s Rehabilitation, Zaozhuang Maternal and Child Health Hospital, Zaozhuang, Shandong Province 277100, P.R. China; 3 Meng Sun, Department of Early Childhood Development Center, Zaozhuang Maternal and Child Health Hospital, Zaozhuang, Shandong Province 277100, P.R. China; 4 Yan Zheng, Department of Pediatrics, Zaozhuang Maternal and Child Health Hospital, Zaozhuang, Shandong Province 277100, P.R. China; 5 Qianqian Zhu, Department of Neonatology, Zaozhuang Maternal and Child Health Hospital, Zaozhuang, Shandong Province 277100, P.R. China; 6 Caixia Liu Department of Neonatology, Zaozhuang Maternal and Child Health Hospital, Zaozhuang, Shandong Province 277100, P.R. China

**Keywords:** Premature infant, Extrauterine growth retardation, First enteral feeding time, Risk factors

## Abstract

**Objectives:**

To understand the incidence and related risk factors of extrauterine growth retardation (EUGR) in preterm infants.

**Methods:**

The clinical data of 132 premature infants hospitalized in the neonatal ward of Maternity and Child Health Care of Zaozhuang from July 2019 to June 2020 were collected. Children were divided into groups according to their birth gestational age (<32 weeks, 32~35 weeks and >35 weeks) and birth weight (<1-500 g, 1,500~2,500 g and >2,500 g). Treatment during hospitalization and follow-up after discharge were investigated by retrospective analysis. Incidence of EUGR in premature infants at discharge and the related risk factors leading to this complication, demonstrated by logistic multivariate regression analysis, were summarized.

**Results:**

When evaluated according to weight, length and head circumference, incidence of EUGR at discharge in premature infants were 36.36%, 41.67% and 21.97% respectively. Smaller gestational age and lower birth weight significantly correlated with higher incidence of EUGR at discharge (P<0.05). Logistic regression analysis showed that small gestational age, low birth weight, intrauterine growth retardation, late first intestinal feeding, digestive system complications and respiratory system complications were independent risk factors for EUGR in premature infants discharged from hospital (P<0.05).

**Conclusions:**

The incidence of EUGR in premature infants at discharge is relatively high. Strengthening perinatal health care for pregnant women, reducing the incidence of intrauterine growth retardation and preterm birth, giving intestinal feeding as soon as possible after birth, and actively participating in preventing and treating postnatal complications are effective ways to reduce the incidence of EUGR at discharge.

## INTRODUCTION

With the continuous improvement of medical technology, the survival rate of very low birth weight infants and preterm infants has significantly improved in recent years.^1^ Due to the complete or partial loss of the fastest intrauterine growth stage, the nutritional reserve in the fetal period is relatively insufficient. Many organs, especially the respiratory, gastrointestinal and immune systems, are not fully developed and mature after birth and are prone to serious complications.^2^ In addition, various diseases associated with prematurity may increase infants’ body metabolism. Therefore, the body heat intake is often not satisfactory, increasing susceptibility to extrauterine growth retardation (EUGR).

At present, EUGR has become a common complication in preterm infants. Studies, evaluating weight, head circumference, body length and other related growth parameters of these children show that they fail to reach the 10th percentile of newborns of the same gestational age.^3^ EUGR not only significantly reduces the early cure rate of preterm infants, but also have a serious impact on their subsequent growth, development and quality of life. EUGR can lead to short stature, mental retardation and poor motor coordination, resulting in a significant burden on the family of the affected children.^4^ The results of follow-up investigations show that, compared with premature infants that match their gestational age at discharge, EUGR premature infants have higher incidence of iron absorption disorder at discharge and the increased incidence risk of nervous system retardation and anemia.^5^,^6^

Some animal studies show that the early occurrence of EUGR can seriously affect the development of pulmonary vessels in preterm infants. This may cause pulmonary hypertension and bronchopulmonary dysplasia, significantly reduce the cure rate of preterm infants, significantly increase the risk of related respiratory diseases after discharge, and the later quality of life.^7^ Recent clinical studies focused on in-depth research of the occurrence of EUGR in preterm infants, its main causes and relevant risk factors in attempt to identify effective methods or means to reduce the incidence of this complication.^8^ The main goal of this retrospective study was to analyze the incidence and related risk factors of extrauterine growth retardation (EUGR) in preterm infants using relevant clinical data of 132 cases of EUGR preterm infants at discharge.

## METHODS

Clinical data and follow-up data of 132 premature infants treated in the neonatal ward Maternity and Child Health Care of Zaozhuang from January 2019 to June 2020 were collected and retrospectively analyzed. The ethics committee of Maternity and Child Health Care of Zaozhuang approved this study (No. 2020068, Date: June 21, 2020).

### Inclusion criteria

Gestational age do not exceed 37 weeks; entry into the neonatal department for treatment within 24 hours after birth, and length of hospital stay not less than seven days; satisfactory relevant clinical data during hospitalization; telephone follow-up done after discharge, with the satisfactory follow-up data.

### Exclusion criteria

Death in hospital or after discharge; serious condition at discharge: genetic metabolic diseases and congenital malformations that have a significant impact on normal growth and development. Patients were divided into groups according to relevant data during hospitalization and follow-up data after discharge.

The data collected included the general information (gender, birth weight and gestational age), postnatal nutritional support, clinical diagnosis and treatment, IUGR occurrence. Relevant information (mainly the feeding status and body mass growth of the children) and data were extracted from weekly telephone follow-up post-discharge up until 44 weeks of corrected gestational age. The main factors related to the occurrence of EUGR were then analyzed.

### Indications for enteral and parenteral nutrition in preterm infants

All children were given formula milk for preterm infants after birth with the volume of milk gradually increased according to the actual condition of infant. Briefly, according to the doctor’s advice, 12ml/kg per day was taken as the initial milk volume. Infants were fed once/every two hours, and the milk volume was gradually increased to 24ml/(kg·d) by the 10th day after birth. From the 11th day after birth, the feeding rate increased from 15~20ml/(kg·d) to 140~160ml/(kg·d). For infants whose growth and development cannot be well met by oral gastrointestinal feeding, parenteral nutrition was given daily according to their required heat card.

### Evaluation criteria of growth retardation

Fenton growth curve for preterm infants was used as the evaluation criteria of IUGR and EUGR at discharge. Neonates were classified as IUGR if their birth weight was below the 10th percentile Compared with newborns of the same gestational age, the body mass at discharge, corrected gestational(P10). EUGR was defined in a static way (weight at discharge below P10 Fenton) and dynamically (decrease over one standard deviation between birth and hospital discharge using Fenton). The percentile reference values of body length, weight, and head circumference of newborns of the same gestational age in 15 cities of China were used as the reference standard. Catch-up growth refers to a phenomenon of accelerated growth of children with growth retardation caused by disease status after removing disease factors.^9^

By analyzing the data, children were divided into groups according to their birth gestational age: gestational age <32 weeks, 32~35 weeks and >35 weeks and weight (<1-500 g, 1,500~2,500 g and >2,500 g). The incidence of EUGR at discharge was compared and analyzed, and multivariate logistic regression analysis was performed.

Statistical analysis was performed by SPSS 22.0 software. All measurement data are expressed in (± s) and for t-test; The counting data is expressed as a percentage “n (%)” and analyzed Χ^2^ inspection. The risk factors were analyzed by logistic regression analysis. When *p<*0.05, the difference was considered statistically significant.

## RESULTS

A total of 132 preterm infants (72 males and 60 females) were included in the study. The average gestational age of the infants was (33.10±2.10) weeks, average birth weight was (2.10±0.35) kg, average length of hospital stay was (18.89±2.10) days, and the average corrected gestational age at discharge was (36.65±1.06) weeks.

The analysis of data showed 43 children with gestational age <32 weeks, 61 children with gestational age of 32~35 weeks and 28 children with gestational age >35 weeks. According to weight, length and head circumference, EUGR was diagnosed in 48 (36.36%) preterm infants in the <32 weeks group, 55 (41.67%) infants in the 32~35 weeks group, and 29 (21.97%) infants in the >35 weeks group (Table-I). As summarized in Table-I, there was a significant correlation between the incidence of EUGR and the weight, length and head circumference of preterm infants of different birth ages (*P<*0.05).

**Table-I:** Comparison of the occurrence of EUGR in children with different gestational ages post-discharge.

Gestational age group at birth	n	EUGR occurs at discharge		

		Weight (48 cases)	Length (55 cases)	Head circumference (29 cases)
<32 weeks group	43	25 (58.14)	24 (55.81)	16 (37.21)
32~35 weeks group	61	18 (29.51)	26 (42.62)	11 (18.03)
>35 weeks group	28	5 (17.86)	5 (17.86)	2 (7.14)
Χ^2^	-	14.195	10.094	9.968
P	-	0.001	0.006	0.007

Among 132 infants in the study, 16 children had birth weight <1500 g, 93 children were with birth weight between 1500~2500 g and 23 children- with birth weight >2500 g. There was a significant association between EUGR and weight, body length and head circumference of infants with different birth weight (*P<*0.05) as shown in Table-II.

**Table-II:** Comparison of the occurrence of EUGR in children with different birth weights post-discharge.

Birth reorganization	n	EUGR occurs at discharge		

		Weight (48 cases)	Length (55 cases)	Head circumference (29 cases)
<1 500 g group	16	11 (68.75)	10 (62.50)	7 (43.75)
1 500~2 500 g group	93	33 (35.48)	41 (44.09)	21 (22.58)
>2 500 g group	23	4 (17.39)	4 (17.39)	1 (4.35)
Χ^2^	-	10.861	8.658	8.614
P	-	0.004	0.013	0.013

Infants were then divided into EUGR group (n=48) and non-EUGR (n=84) groups according to their weight, and gestational age, length of hospital stay and related complications between the two groups were compared. As shown in Table-III, birth gestational age and birth weight of children in the EUGR group were significantly lower than in the non EUGR group (P<0.001). Infants in the EUGR group had higher incidence of IUGR compared to non-EUGR group (P<0.001). Age of first intestinal feeding in the EUGR group was lower than in non-EUGR group (P<0.001), and the time of first gastrointestinal nutrition was significantly delayed compared to non-EUGR group (P<0.001). There was markedly higher incidence of respiratory complications in the EUGR group as compared to non-EUGR group (P<0.05).

**Table-III:** Comparison of basic data of children in EUGR group and non-EUGR group.

Factor	Non-EUGR group(n=84)	EUGR group(n=48)	Χ^2^/t	P
Male/female (example)	47/37	25/23	0.184	0.668
Gestational age (±s, weeks)	33.83±1.96	31.81±1.70	6.220	<0.001
Birth weight (±s, g)	2180.84±372.21	1958.33±265.18	3.987	<0.001
Hospitalization time (±s, d)	18.63±1.73	19.35±2.60	1.722	0.089
Birth weight recovery time (±s, d)	10.48±2.66	11.27±2.09	1.870	0.064
Incidence rate of IUGR[n(%)]	9(10.71)	25(52.08)	27.336	<0.001
Complications during pregnancy[n(%)]	24(28.57)	15(31.25)	0.105	0.746
Age of first enteral feeding (±s, d)	1.35±1.23	2.07±0.18	4.071	<0.001
Time for total gastrointestinal nutrition (±s, d)	11.92±2.00	14.81±2.21	7.699	<0.001
Digestive system	3(3.57)	8(16.67)	6.857	0.009
Respiratory system	8(9.52)	11(22.92)	4.446	0.035
Blood system	20(23.81)	13(27.08)	0.175	0.676
Cardiovascular System	10(11.90)	5(10.42)	0.067	0.796
Urinary system	3(3.57)	1(2.08)	0.230	0.631
nervous system	13(15.48)	7(14.58)	0.019	0.891
Metabolic disorders	65(77.38)	43(89.58)	3.057	0.080

Logistic regression analysis was next performed with EUGR at discharge as the dependent variable and birth gestational age, birth weight, IUGR, time of first intestinal feeding, complications of digestive system and respiratory system as independent variables. As shown in Table-IV, small gestational age, low birth weight, intrauterine growth retardation, late first intestinal feeding and digestive and respiratory diseases were all independent risk factors for EUGR in premature infants (P<0.05).

**Table-IV:** Logistic regression analysis of risk factors for EUGR when premature infants post-discharge.

Variable	B	S.E.	Wald	df	P	OR	95%CI
Gestational age at birth	-0.77	0.286	7.221	1	0.007	0.463	0.264~0.812
Birth weight	0.004	0.002	5.007	1	0.025	1.004	1.000~1.007
IUGR	1.971	0.626	9.902	1	0.002	7.177	2.103~24.495
Age of first enteral feeding	0.399	0.218	3.328	1	0.068	1.490	0.971~2.286
Time for total gastrointestinal nutrition	0.921	0.243	14.367	1	P<0.001	2.511	1.560~4.043
Digestive system	2.206	1.076	4.203	1	0.04	9.080	1.102~74.824
Respiratory system	1.99	0.797	6.228	1	0.013	7.315	1.533~34.911

## DISCUSSION

EUGR not only affects the short-term physical development and incidence of related complications of newborns, but also have a serious impact on their long-term growth and development, especially the development of nervous system.^10^ It can negatively affect metabolic indexes and blood pressure levels of children in early adolescence, thus increasing the risk of metabolic syndrome and cardiovascular disease.^11^,^12^ At present, numerous studies show that intrauterine growth retardation, low birth weight and small birth age are high- risk factors for EUGR in preterm infants.^13^,^14^ However, some researchers believe that gender, postnatal complications and the first intestinal feeding time are also important factors leading to EUGR in preterm infants.^15^,^16^

The results of this study show a significant correlation between the low gestational age and birth weight and the risk of EUGR. Carducci B et al.^17^ analyzed the association of premature EUGR with gestational age ≤ 34 weeks, and showed that the proportion of premature infants with birth weight <1500 g and gestational age <28 weeks in the delayed group was significantly higher than that in the control group. EUGR is defined as a varying degree of hindered growth and development during the first few weeks after birth due to severe malnutrition. Although there is a phenomenon of “catch-up growth” within two months after birth, most children with EUGR show significantly less growth and development indicators after discharge, such as chest circumference, head circumference and weight, compared with the corresponding expected value of intrauterine growth rate.^18^

There are many reasons for the occurrence of EUGR after discharge of preterm infants. Darmun D et al.^19^ showed that compared with normal preterm infants, preterm infants in an EUGR group have low birth weight, small gestational age, high proportion of intrauterine growth retardation. EUGR was also associated with high proportion of pregnant women with pregnancy complications. These findings are in agreement with the results of our study.

### Limitation of the study

The main limitation of this study is that all data was acquired from a single center retrospectively and relied on accurate, detailed and available preterm data. Further large-scale and multi-center studies are needed to confirm our observations.

## CONCLUSION

The risk of EUGR in preterm infants is high. The independent risk factors leading to this complication are small gestational age, low weight, intrauterine growth retardation, late first enteral feeding, digestive system diseases and respiratory diseases. This study may provide reference for the prevention of EUGR in preterm infants.

### Authors’ contributions:

**MS:** Conceived and designed the study. **JL, MS, YZ, QZ & CL:** Collected the data and performed the analysis. **MS:** Was involved in the writing of the manuscript and is responsible for the integrity of the study. All authors have read and approved the final manuscript.
